# Efficacy and Safety of Anlotinib in the Treatment of Small Cell Lung Cancer: A Real-World Observation Study

**DOI:** 10.3389/fonc.2022.917089

**Published:** 2022-06-20

**Authors:** Jing Yuan, Feng Cheng, Guodong Xiao, Xiaofeng Wang, Huijie Fan

**Affiliations:** Department of Oncology, The First Affiliated Hospital of Zhengzhou University, Zhengzhou, China

**Keywords:** small cell lung cancer, anlotinib, maintenance therapy, second-line treatment, third-line treatment

## Abstract

**Aims:**

This study aimed to observe the efficacy and safety of anlotinib in the treatment of small cell lung cancer (SCLC) in the real world, as first-line maintenance therapy, second-line, and above.

**Methods:**

Clinical data of 109 patients with SCLC treated with anlotinib and hospitalized at The First Affiliated Hospital of Zhengzhou University from June 2018 to June 2020 were retrospectively analyzed. Analysis of short-term efficacy and survival was performed, with *p*<0.05 being considered statistically significant.

**Results:**

The median progression-free survival (mPFS) of anlotinib monotherapy used as first-line maintenance treatment of SCLC was 6.3 months (11.7 months in the limited phase and 5.8 months in the extensive phase) and median overall survival (mOS) was 16.7 months (not reached in limited phase, 12.6 months in extensive phase). In second-line treatment, anlotinib with chemotherapy prolonged PFS and OS as compared to anlotinib monotherapy (*p*<0.05). In third-line and above treatment, there was no improvement in mPFS with the chemotherapy combination regimen compared to anlotinib monotherapy (3.6 months vs. 3.8 months, *p=0*.398), with a trend toward impaired mOS (8.5 months vs. not achieved, *p=0*.060). Univariate analyses and multivariate analyses revealed that Eastern Cooperative Oncology Group performance status and liver metastases were independent prognostic factors affecting PFS and OS. No new anlotinib-related adverse reactions were identified.

**Conclusion:**

Anlotinib was effective for first-line maintenance and second-line treatment, and the chemotherapy combination regimen was superior to monotherapy when applied as second-line treatment. However, this trend was not observed in third-line and above therapy.

## Introduction

In recent years, among all cancers, lung cancer has the highest incidence and mortality rate worldwide (1). Small cell lung cancer (SCLC) is an aggressive neuroendocrine tumor, which accounts for approximately 15% of all lung cancers, and its biological and clinical characteristics are completely different from other types of lung cancers. SCLC is more sensitive to chemotherapy, but it easily develops resistance to chemotherapeutic drugs due to the rapid proliferation rate of the tumor. As a result, most patients experience disease progression soon after first-line treatment, and the efficacy of subsequent treatments is low. While the survival of patients with SCLC has improved over the years (2, 3), more safe and efficacious drugs are still urgently needed.

Anlotinib is a multi-targeted small molecule tyrosine kinase inhibitor (TKI) developed independently in China. In its phase II clinical trial ALTER1202, anlotinib was found to significantly prolong progression-free survival (PFS) and overall survival (OS) in third-line and above treatment of SCLC compared with a placebo. The Chinese treatment guidelines for SCLC have since been revised to reflect this new treatment (4). However, in practice, anlotinib is used not only in third-line and above therapies in the treatment of SCLC, but also in first-line maintenance therapy and second-line treatment. In terms of usage, it is rather common to combine chemotherapy, immunotherapy, or other treatments. However, there are few studies on the application of anlotinib in SCLC at the frontline. Therefore, this study aimed to analyze real-world data on anlotinib to evaluate whether anlotinib could provide additional benefits to SCLC patients as first-line maintenance therapy and second-line treatments, as well as whether combination therapy with chemotherapy or immune checkpoint inhibitors (ICIs) could further improve its efficacy.

## Materials and Methods

### Clinical Data

**Figure 1:**
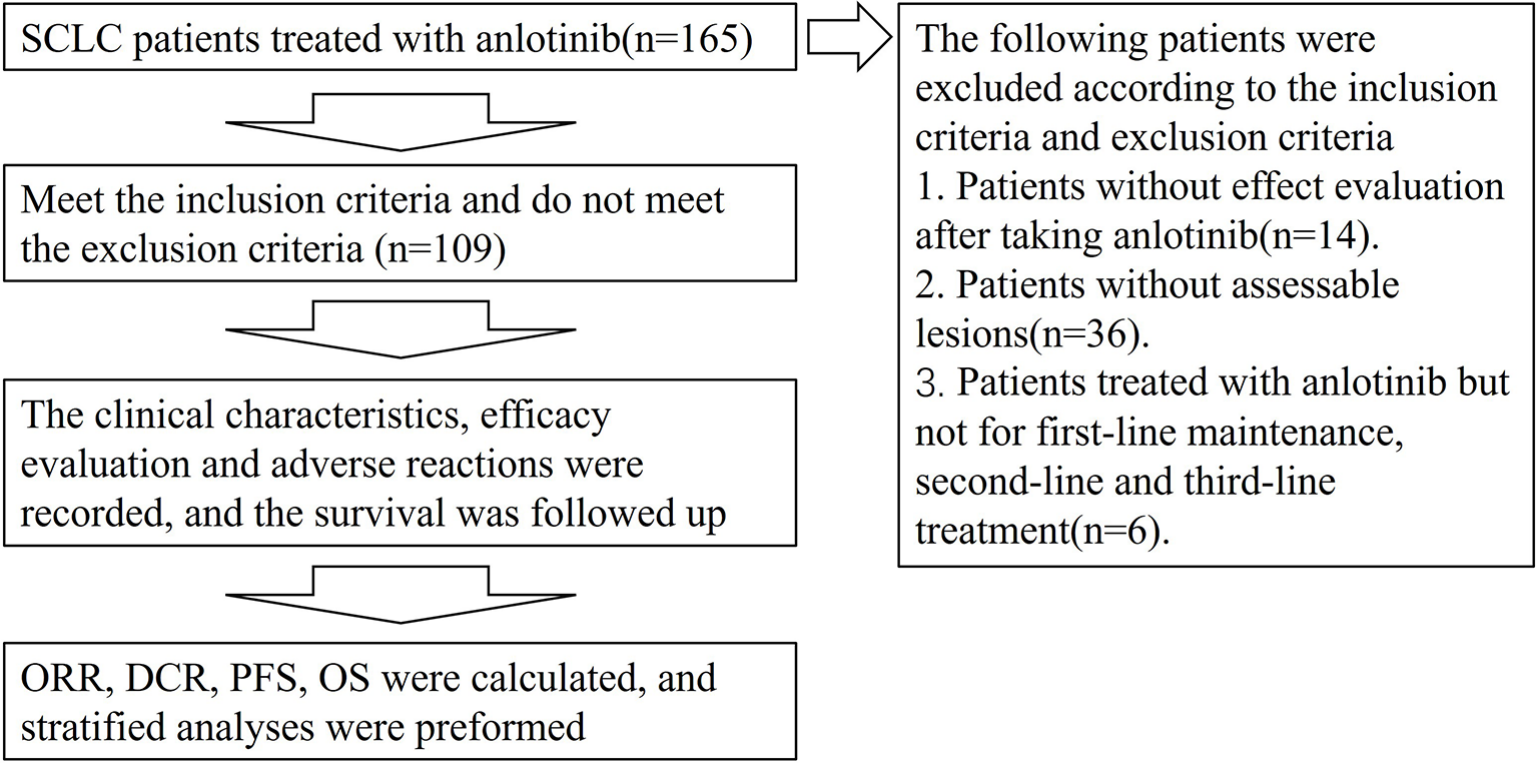
Flow chart.

Clinical data of patients with SCLC, treated with anlotinib hospitalized at The First Affiliated Hospital of Zhengzhou University from June 2018 to June 2020, were collected. The main inclusion criteria included: (1) pathologically confirmed SCLC; (2) patients with SCLC receiving first-line maintenance therapy with anlotinib or as second- or third-line after progression on prior therapies and subsequent treatments; (3) at least one observable or measurable lesion according to the Response Evaluation Criteria in Solid Tumors (RECIST) guidelines version 1.1. Those cases with no follow-up data after treatment with anlotinib were excluded. Based on the above criteria, 109 patients with SCLC were included. The study workflow is outlined in **Figure 1**.

### Treatment Methods

Anlotinib capsules (Chia Tai Tianqing Pharmaceutical Group Co., Ltd., Approval No.: National Drug Code H20180002) were prescribed in an 8–12 mg dose and taken orally once a day for 2 weeks with a 1-week break. The dose prescribed depended on the patient’s age, ECOG PS, and body surface area. The dose could be titrated downwards according to the patient’s tolerance post-administration.

### Evaluation of Efficacy and Observation Metrics

RECIST version 1.1 was used to evaluate the efficacy of the treatment. Responses were classified as complete response (CR), partial response (PR), stable disease (SD), and progressive disease (PD). The objective response rate (ORR) was calculated as the proportion of patients achieving CR or PR. The disease control rate (DCR) is the proportion of patients who achieved CR, PR, or SD. PFS is defined as the period from when a patient starts oral anlotinib until disease progression or death from any cause. OS is defined as the time from the start of patients receiving anlotinib until death due to any cause. For subjects who were lost to follow-up prior to death, the time of the last follow-up visit was recorded as the time of death.

The classification of drug-related adverse reactions was evaluated and recorded according to the Common Terminology Criteria for Adverse Events (CTCAE) version 4.03.

### Follow-Up Visits

The final follow-up visit was conducted up to December 19, 2020, mainly by telephone and outpatient follow-up appointments. The time to disease progression or death was recorded. For the end of follow-up, cases with a follow-up outcome of “loss to follow-up” were considered as censored cases. There were 15 cases lost to follow-up in this study, with a 13.8% loss to follow-up rate and a median follow-up time of 16.7 months (95% CI: 10.3 months to 23.1 months).

### Statistical Methods

SPSS version 18.0 was used for statistical analysis. The chi-square test was used for rate comparison, the Kaplan–Meier method was used for survival analysis, log-rank test was used to compare survival time between different groups, and the Cox regression method was used for multivariate analysis. A *p*-value of <0.05 was considered statistically significant.

## Results

### Basic Patient Information

Clinical data were collected from 109 patients with SCLC treated with anlotinib. Information on age, sex, smoking history, ECOG PS, clinical staging, metastatic site, combination therapy with anlotinib, therapeutic drugs before and after anlotinib administration, and whether radiotherapy was done was collected. Detailed information is shown in **Table 1**.

**Table 1:** Patient characteristics at baseline (n=109 cases).

Characteristic	Groups	Value (%)
Gender	Male	79 (72.5%)
	Female	30 (27.5%)
Age	≤65 years	74 (67.9%)
	>65 years	35 (32.1%)
Smoking history	Yes	56 (51.4%)
	No	53 (48.6%)
ECOG PS	0-1	94 (86.2%)
	≥2	15 (13.8%)
Disease extent	Limited	38 (34.9%)
	Extensive	71 (65.1%)
Brain metastases	Yes	31 (28.4%)
	No	78 (71.6%)
Liver metastases	Yes	11 (10.1%)
	No	98 (90.0%)
Treatment lines	Maintenance therapy after 1^st^ line	35 (32.1%)
	2^nd^ line	39 (35.8%)
	≥3^rd^ line	35 (32.1%)
Ki67 index	≥90%	58 (53.2%)
	<90%	42 (38.5%)
	unknown	9 (8.3%)
PFS of 1st-line therapy	≤3 months	8 (7.3%)
	>3 months	101 (92.7%)
ICIs treatment	Yes	35 (32.1%)
	No	74 (67.9%)
Previous radiation therapy	Yes	59 (54.1%)
	No	50 (45.9%)
Previous antiangiogenic treatment	Yes	16 (14.7%)
	No	93 (85.3%)

### Short-Term Efficacy

Treatment efficacy was evaluated for all 109 patients enrolled in this study. A total of 35 patients received anlotinib monotherapy as first-line maintenance. A total of 39 patients received anlotinib as second-line treatment with an overall ORR of 17.9% and DCR of 76.9%. Sixteen out of 39 patients received anlotinib monotherapy and 23 were treated with other drugs in combination (including chemotherapy, ICIs, and chemotherapy with ICIs). The ORR and DCR of combination therapy (ORR, 26.1%; DCR, 87%) were improved in comparison with monotherapy (ORR, 6.25%; DCR, 62.5%). However, there was no statistically significant difference (*χ* ^2^ value = 4.273, *p*=0.119). A total of 35 patients received anlotinib as third- and further-line treatment, with an overall ORR of 17.1% and an overall DCR of 85.7%. Twelve of the patients received anlotinib monotherapy, and 23 patients were treated in combination with other treatments (including chemotherapy, ICIs, and local treatment). The ORR and DCR of the combination therapy (ORR, 21.7%; DCR, 87%) were not statistically significant (*χ* ^2^ value = 1.010, *p*=0.750), as detailed in **Table 2**.

**Table 2:** Observation of short-term efficacy.

Treatment lines	Cases(n)	Combination therapy	PR	SD	PD	ORR	DCR	Total ORR	Total DCR
2st line	16	None	1	9	6	6.25%	62.5%	17.9%	76.9%
	16	Chemotherapy	2	12	2	12.5%	87.5%		
	5	ICIs	3	1	1	60%	80%		
	2	Chemothrapy & ICIs	1	1	0	50%	100%		
≥3rd line	12	None	1	9	2	8.3%	83.3%	17.1%	85.7%
	14	Chemotherapy	2	11	1	14.3%	92.9%		
	8	ICIs	3	3	2	37.5%	75%		
	1	Interventional therapy	0	1	0		100%		

### Survival Analysis

At the end of follow-up, 78 (71.6%) patients reached disease progression on anlotinib at their last follow-up, with an overall median PFS (mPFS) of 6.3 months. Forty-three patients reached the endpoint of death at their last follow-up, accounting for 39.4% of the total. The overall median OS (mOS) was 10.3 months.

**Figure 2:**
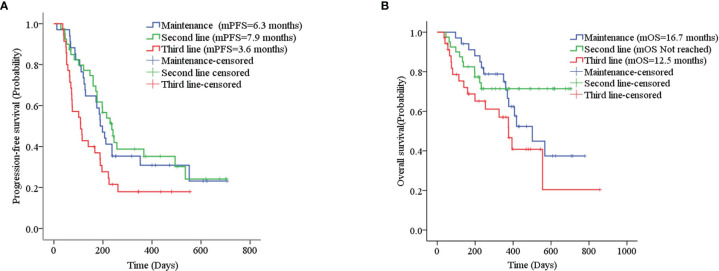
**(A)** Kaplan–Meier plot of PFS among different treatment lines; **(B)** Kaplan–Meier plot of OS among different treatment lines.

Thirty-five patients with SCLC receiving anlotinib as first-line maintenance therapy (17 patients in the limited phase and 18 patients in the extensive phase) had an mPFS of 6.3 months (11.7 months in the limited phase and 5.8 months in the extensive phase) and an mOS of 16.7 months (not reached in the limited phase and 12.6 months in the extensive phase), as shown in **Figures 2A, B** and **Supplemental Figure 1A, B**.

**Figure 3:**
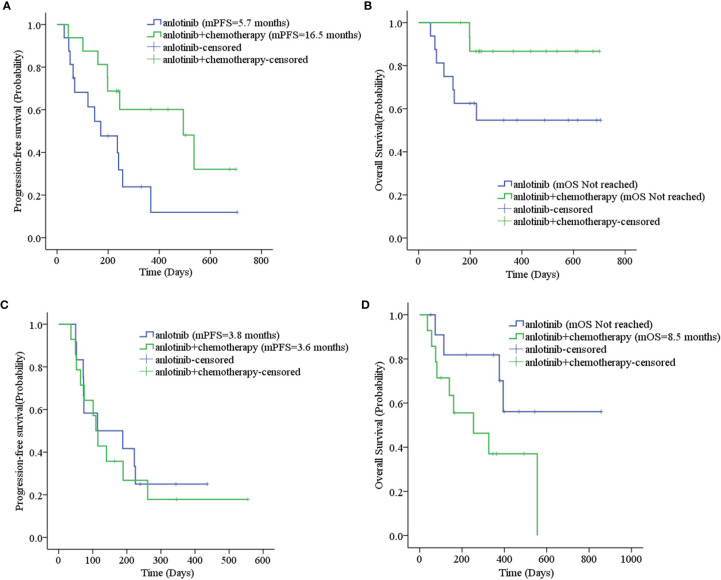
**(A)** Progression-free survival stratified by treatment regimen in second-line therapy; **(B)** Overall survival stratified by treatment regimen in second-line therapy; **(C)** Progression-free survival stratified by treatment regimen in third- and further-line therapy; **(D)** Overall survival stratified by treatment regimen in third- and further-line therapy.

The mPFS was 7.9 months for second-line treatment with anlotinib and 3.6 months for third-line and above therapy, as shown in **Table 3** and **Figure 2A**. In second-line treatment, mPFS was 5.7 months for the anlotinib monotherapy group (Group A) and 16.5 months for the anlotinib-chemotherapy combination group (Group A+C), with statistically significant difference (*χ* ^2 =^ 4.208, *p=0*.04). In third-line and above therapy, mPFS for Group A and Group A+C were 3.8 months and 3.6 months, respectively, with no statistically significant difference (*χ* ^2^ = 0.138, *p=0*.711), as shown in **Figures 3A, C**.

**Table 3:** Univariate survival analysis.

Characteristic	Groups	mPFS (m)	95%CI	*χ^2^* value	*P value*	mOS (m)	95%CI	*χ^2^* value	*P value*
Gender	Male	6.3	5.6-7.0	0.002	0.969	16.7	3.8-24.1	0.771	0.38
	Female	5.6	2.3-8.9			18.5	NR		
Age	≤65	6.2	5.3-7.1	0.411	0.522	18.9	NR	0.011	0.918
	>65	6.6	4.8-8.5			18.5	9.9-27.1		
Smoking history	Yes	6.2	5.2-7.1	0.085	0.77	18.9	NR	0.029	0.864
	No	6.6	4.9-8.3			16.7	9.8-23.6		
ECOG PS	0-1	6.9	5.8-8.0	22.825	**<0.001**	NR	NR	36.972	**<0.001**
	≥2	2.3	1.8-2.7			4.6	2.4-6.8		
Disease extent	Limited	7.6	4.5-10.7	1.921	0.166	18.9	NR	0.877	0.349
	Extensive	6.2	5.3-7.1			16.7			
Brain metastases	Yes	6.3	4.0-8.5	0.049	0.824	NR	NR	2.053	1.052
	No	6.2	5.0-7.3			16.7	10.1-23.3		
Liver metastases	Yes	3.6	1.7-5.5	13.325	**<0.001**	6.6	3.8-9.4	27.415	**<0.001**
	No	6.6	5.5-7.7			NR	NR		
Treatment lines	Post 1^st^ line maintenance	6.3	5.3-7.3	7.43	**0.024**	16.7	11.3-22.1	5.88	0.053
	2^nd^ line	7.9	6.2-9.6	5.377		NR	NR	4.329	
	≥3 line	3.6	2.1-5.2			12.5	9.8-15.3		
Ki67 index	≥90%	5.9	4.8-6.9	0.19	0.663	16.7	9.6-23.8	0.768	0.381
	<90%	6.3	4.9-7.6			NR	NR		
PFS of 1st-line therapy	≤3 months	6.3	4.0-8.6	0.523	0.47	12.5	NR	0.014	0.906
	>3 months	6.3	5.5-7.1			18.5	NR		
ICIs treatment	Yes	5.9	2.7-9.1	1.659	0.198	12.5	3.8-21.2	2.373	0.123
	No	6.3	5.1-7.5			18.9	NR		
Previous radiation therapy	Yes	6.3	5.8-6.8	0.137	0.712	NR	NR	1.019	0.313
	No	5.7	2.7-8.7			13.9	8.6-19.3		
Previous antiangiogenic treatment	Yes	3.8	0.0-10.0	3.766	0.052	10.9	4.7-17.1	3.821	0.051
	No	6.3	5.3-7.3			18.9	NR		

CI, Confidence Interval; NR, Not Reached. P value less than 0.05 were shown in bold.

The mOS of anlotinib, when administered in second-line treatment, was better than that of third-line and above therapy (*p=0*.037), as shown in **Table 3** and **Figure 2B**. In second-line treatment, mOS was not achieved in both the monotherapy and combination groups. The mOS was better in Group A+C than in Group A (*χ* ^2 =^ 4.214, *p=0*.040). In third-line and above therapy, mOS was not achieved in Group A, and was 8.5 months in Group A+C (*χ* ^2 =^ 3.027, *p=0*.082), as shown in **Figures 3B–D**.

**Figure 4:**
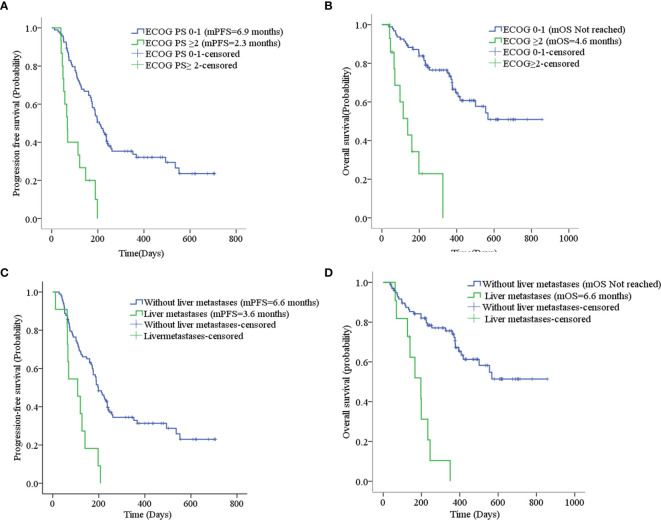
**(A)** Progression-free survival stratified by ECOG PS; **(B)** Overall survival stratified by ECOG PS; **(C)** Progression-free survival stratified by liver metastases; **(D)** Overall survival stratified by liver metastases.

Sixteen patients who had received other anti-angiogenic treatments (including endostatin, bevacizumab, and apatinib) showed some difference in mPFS from those who had not received anti-angiogenic treatment (3.8 months vs. 6.3 months, *p*=0.052), as shown in **Table 3**. Considering that only one patient had received anti-angiogenic treatment (apatinib) with first-line therapy, we excluded the 35 patients in the first-line maintenance portion when evaluating the effect of anti-angiogenic agents on the efficacy of anlotinib. The PFS was 5.6 months for other patients who had previously used anti-angiogenic therapy compared to 6.3 months for those who had not, with no statistically significant difference (χ^2^ = 1.936, *p=0*.164). There was also no statistically significant difference in mOS (*χ* ^2^ = 2.215, *p=0*.137), as shown in **Supplemental Figures 2A, B**. Eighteen patients were treated by ICIs previously or concomitantly with anlotinib. However, there were no significant differences in PFS (*χ* ^2^ = 1.659, *p=0*.198) and OS (*χ* ^2^ = 2.373, *p=0*.123) according to ICI treatment, as shown in **Table 3** and **Supplemental Figures 2C, D**. Patients with liver metastases and an ECOG PS score ≥2 had worse PFS and OS than the corresponding control group (*p*<0.001), as shown in **Table 3** and **Figure 4**. Cox regression analysis indicated that patients with ECOG PS ≥2 or liver metastases had a shorter PFS and OS (*p*<0.001), as shown in **Table 4**.

**Table 4:** Cox regression analysis of PFS and OS.

Groups	PFS			OS		
	*p* value	HR	95%CI	*p* value	HR	95%CI
Liver metastases: Yes vs No	<0.001	3.769	1.921-7.391	<0.001	9.622	4.203-22.024
ECOG PS: ≥2 vs 0-1	<0.001	3.823	2.027-7.208	<0.001	9.968	4.232-23.479
Treatment lines: ≥3rd line vs 2nd line vs Post 1st line maintenance	0.145	1.258	0.924-1.713	0.225	1.288	0.856-1.936

### Evaluation of Drug Safety

Incidences of adverse reactions, such as fatigue, hand and feet reactions, hypertension, elevated ALT/AST, loss of appetite, and proteinuria, during the use of anlotinib were greater than 20%, regardless of severity grade. The incidence of grade 3 and above adverse reactions was 20.2%, and a total of four patients discontinued anlotinib due to intolerable adverse reactions (hemoptysis, elevated ALT/AST, severe thrombocytopenia, and joint pain), as shown in **Table 5**.

**Table 5:** Incidence of adverse reactions.

Adverse reaction	Any grade (%)	Grade 3/4 (%)
Fatigue	51 (46.8%)	6 (5.5%)
Hand-foot syndrome	41 (37.6%)	2 (1.83%)
Hypertension	35 (32.1%)	2 (1.83%)
ALT/AST elevation	28 (25.7%)	3 (2.8%)
Loss of appetite	26 (23.9%)	3 (2.8%)
Proteinuria	23 (21.1%)	1 (0.9%)
Hemoptysis	17 (15.6%)	3 (2.8%)
Oral mucositis	12 (11.0%)	0 (0%)
Thrombocytopenia	10 (9.2%)	1 (0.9%)
Thrombotic events	3 (2.8%)	1 (0.9%)
Joint pain	2 (1.83%)	0 (0%)
Prolonged Q-T interval	1 (0.9%)	0 (0%)

## Discussion

Angiogenesis is a key component of tumor proliferation and metastasis (5). It was found that vascular endothelial growth factor (VEGF) levels were significantly higher in patients with SCLC than in the healthy population (6), suggesting that anti-angiogenic therapy may be effective in SCLC.

Anlotinib is a small molecule anti-angiogenic drug developed independently in China that inhibits tumor neovascularization by regulating VEGF, fibroblast growth factor, and platelet-derived growth factor receptors. It inhibits tumor growth by inhibiting c-Kit, a target related to tumor proliferation, invasion, and migration. Anlotinib has shown good efficacy and safety in lung cancer, soft tissue sarcoma, kidney cancer, and other cancer types (7–9). In the field of SCLC, the ALTER1202 study comparing the efficacy and safety of anlotinib against placebo for third-line and above treatment of SCLC showed that the anlotinib group had significantly better PFS and OS than the placebo group with a favorable safety profile (4, 10).

Maintenance therapy for SCLC is not as well reported. Trials on bevacizumab as first-line and first-line maintenance therapy for SCLC have been conducted; however, bevacizumab only improved PFS and did not prolong OS (11, 12). Sunitinib, a multi-targeted small molecule TKI, prolonged PFS (mPFS 3.7 months vs. 2.1 months, *p=0*.02), but was poorly tolerated and did not exhibit a significant difference in OS (9.0 months vs. 6.9 months, *p=0*.16) when compared to placebo as maintenance therapy after first-line chemotherapy for extensive stage SCLC (13). Studies have also attempted the use of a single chemotherapeutic agent as a first-line maintenance regimen for treatment, but the choice of irinotecan, topotecan, or etoposide did not significantly improve OS (14–16). In this study, 35 patients receiving anlotinib as first-line maintenance therapy were enrolled and all were treated with etoposide in combination with platinum as first-line chemotherapy. The mPFS for maintenance therapy with anlotinib in extensive stage SCLC was 5.8 months, and the mOS exceeded 1 year at 12.6 months.

Second-line treatment options are relatively limited for SCLC. Topotecan monotherapy is the standard second-line treatment regimen of SCLC (17). The PFS of relapsed SCLC treated with amrubicin or EP regimen rechallenge were 3.5 and 4.7 months respectively, which were better than that of the topotecan control groups (2.2 and 2.7 months respectively) (18, 19). ICIs such as nivolumab did not improve survival when compared with chemotherapy in relapsed SCLC (20, 21). In this study, the mPFS for second-line treatment with an anlotinib-containing regimen was 7.9 months. The PFS and OS of anlotinib-chemotherapy combination were significantly prolonged compared with anlotinib monotherapy. In third-line and above treatment, there was no benefit to PFS and even an impaired OS for the anlotinib-chemotherapy combination as compared to anlotinib monotherapy. Anlotinib-containing regimens may be an alternative for relapsed SCLC. However, the benefit of chemotherapy combination was greater in the second-line application of anlotinib, not in third-line and above applications. Therefore, it appears that different regimen designs should be chosen at different treatment times.

In a univariate analysis, liver metastasis and ECOG PS score were found to be prognostic factors for PFS and OS, which was consistent with previous studies (22). We also found that prior anti-angiogenic therapy did not affect the efficacy of anlotinib. Unfortunately, no synergistic effect of ICIs on anlotinib was observed in the study.

The incidence of common adverse effects and adverse reactions to anlotinib in this study were similar to those reported previously (4), suggesting that although anlotinib was often combined with other therapeutic agents in actual clinical application, there was no significant increase in the incidence of anlotinib-related adverse reactions.

In summary, we found further evidence for the use of anlotinib in first-line maintenance and second-line therapy, providing a new option for relapsed SCLC. In addition, we found that anlotinib had added benefit in combination with chemotherapy in second-line therapy; this was not observed in third-line therapy. This suggests that the formulation of individualized treatment plans will be of great help in improving the efficacy of SCLC treatment. However, the results should be validated by a randomized controlled prospective study.

## Data Availability Statement

The raw data supporting the conclusions of this article will be made available by the authors, without undue reservation.

## Author Contributions

This study was conceived, designed, and interpreted by HF and JY. JY performed the data analyses and drafted the manuscript; FC contributed significantly to analysis and manuscript preparation; GX and XW helped perform the analysis with constructive discussions. All authors read and approved the final manuscript.

## Funding

This study received a specific grant from the Key Scientific Research Project of Higher Education in Henan Province (Project Number: 21A320045, to HF) and Wu Jieping Medical Foundation for clinical Research (Project No: 320.6750.2021-01-8, to HF).

## Conflict of Interest

The authors declare that the research was conducted in the absence of any commercial or financial relationships that could be construed as a potential conflict of interest.

## Publisher’s Note

All claims expressed in this article are solely those of the authors and do not necessarily represent those of their affiliated organizations, or those of the publisher, the editors and the reviewers. Any product that may be evaluated in this article, or claim that may be made by its manufacturer, is not guaranteed or endorsed by the publisher.

## Supplementary Material

Supplementary Figure 1
**(A)** Progression-free survival stratified by clinical stage of SCLC when anlotinib was used as first-line maintenance regimen. **(B)** Overall survival stratified by clinical stage of SCLC when anlotinib was used as first-line maintenance regimen.

Supplementary Figure 2
**(A)** Progression-free survival stratified by previous antiangiogenic treatment; **(B)** Overall survival stratified by previous antiangiogenic treatment; **(C)** Progression-free survival stratified by ICIs treatment; **(D)** Overall survival stratified by ICIs treatment.
