# Supplementary figures and images for: Efficacy and Safety of Anlotinib in the Treatment of Small Cell Lung Cancer: A Real-World Observation Study
Source: Front Oncol. 2022 Jun 20;12:917089. doi: 10.3389/fonc.2022.917089 (PMC9251318; doi:10.3389/fonc.2022.917089)

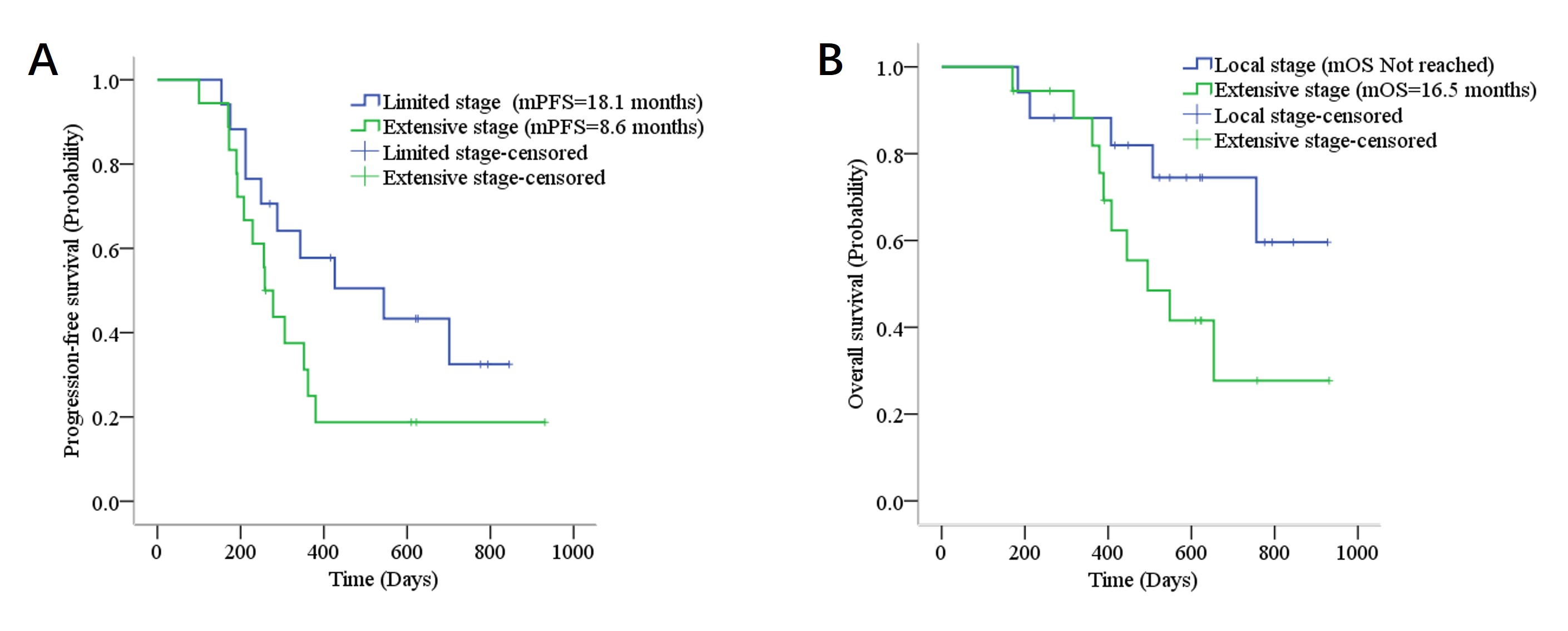

Supplement: Supplementary Figure 1 — (A) Progression-free survival stratified by clinical stage of SCLC when anlotinib was used as first-line maintenance regimen. (B) Overall survival stratified by clinical stage of SCLC when anlotinib was used as first-line maintenance regimen. [file Image_1.jpeg]

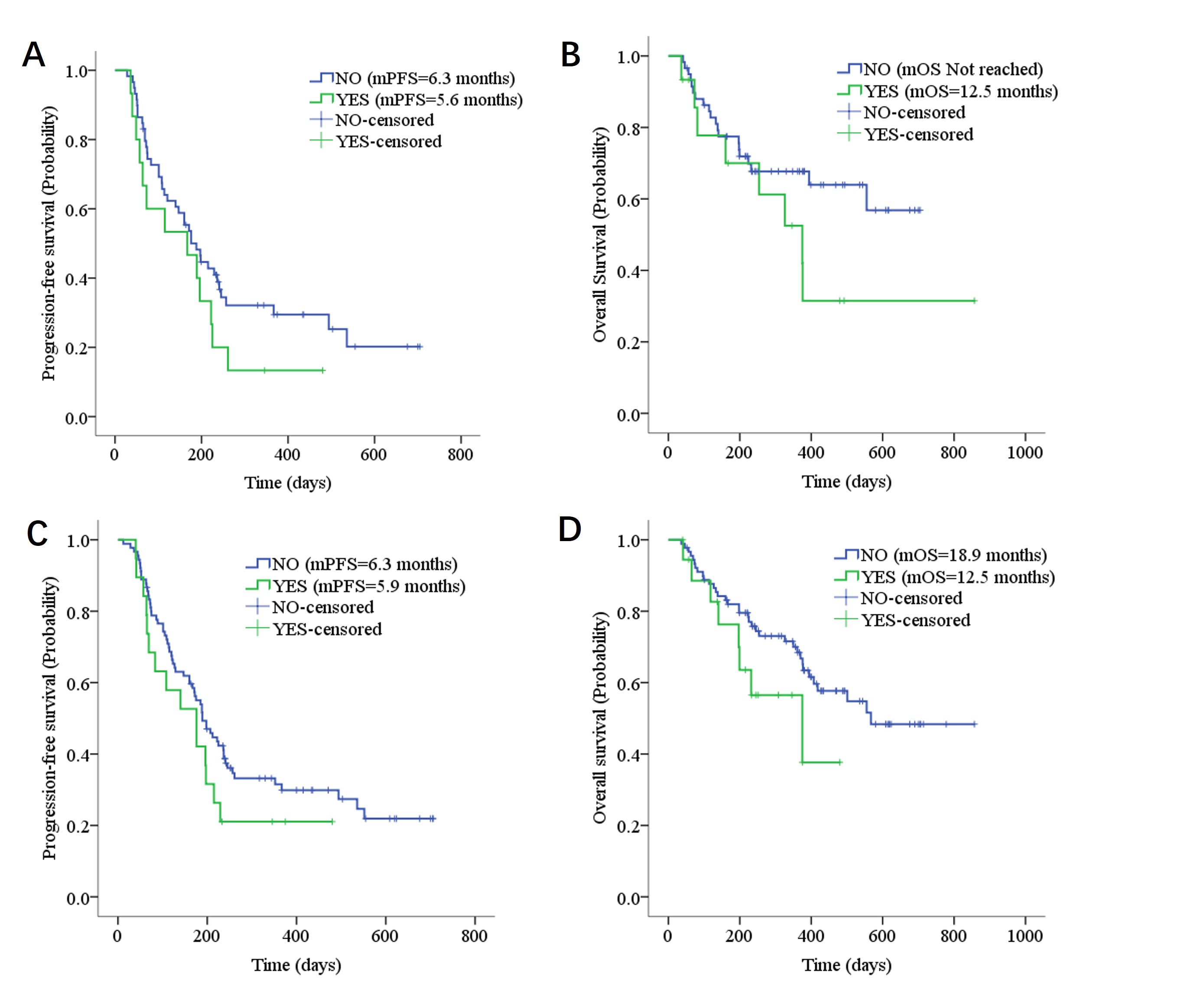

Supplement: Supplementary Figure 2 — (A) Progression-free survival stratified by previous antiangiogenic treatment; (B) Overall survival stratified by previous antiangiogenic treatment; (C) Progression-free survival stratified by ICIs treatment; (D) Overall survival stratified by ICIs treatment. [file Image_2.jpeg]
